# Supplementary material for: Empagliflozin Improves Metabolic and Hepatic Outcomes in a Non-Diabetic Obese Biopsy-Proven Mouse Model of Advanced NASH
Source: Int J Mol Sci. 2021 Jun 13;22(12):6332. doi: 10.3390/ijms22126332 (PMC8232001; doi:10.3390/ijms22126332)
Supplement: Supplementary file 1 [file ijms-22-06332-s001.zip › ijms-1238675-supplementary.pdf]

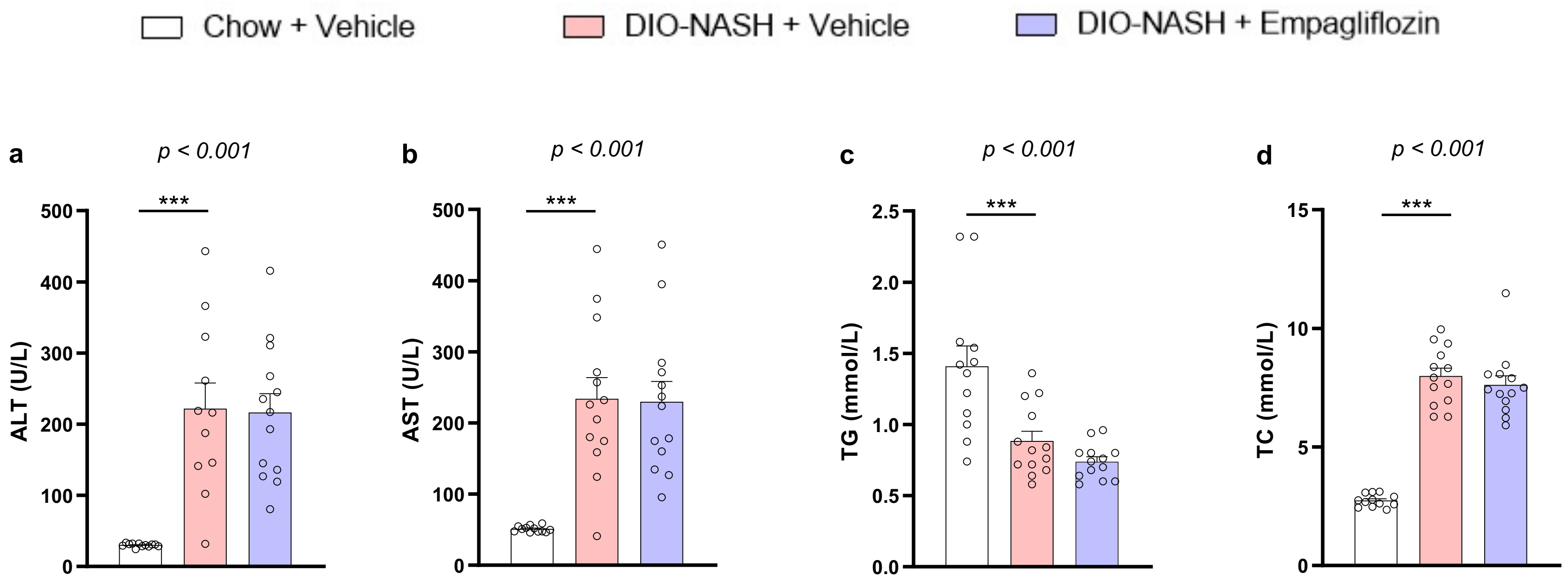

Supplemental Figure 1: Effects of empagliflozin on liver transaminases and blood lipids at treatment completion. (a) ALT, (b) AST, (c) Triglycerides, (d) Total cholesterol. Data show means ± SEMs. By $p < 0.05$ in ANOVA, post-hoc Dunnett tests were performed in each timepoint and *$P < 0.05$, **$P < 0.01$, ***$P < 0.001$, respectively for Chow + vehicle or DIO-NASH + empagliflozin compared to DIO-NASH + vehicle is reported. Chow + vehicle n=12; DIO-NASH +Vehicle n=13; DIO-NASH + Empagliflozin n=13.

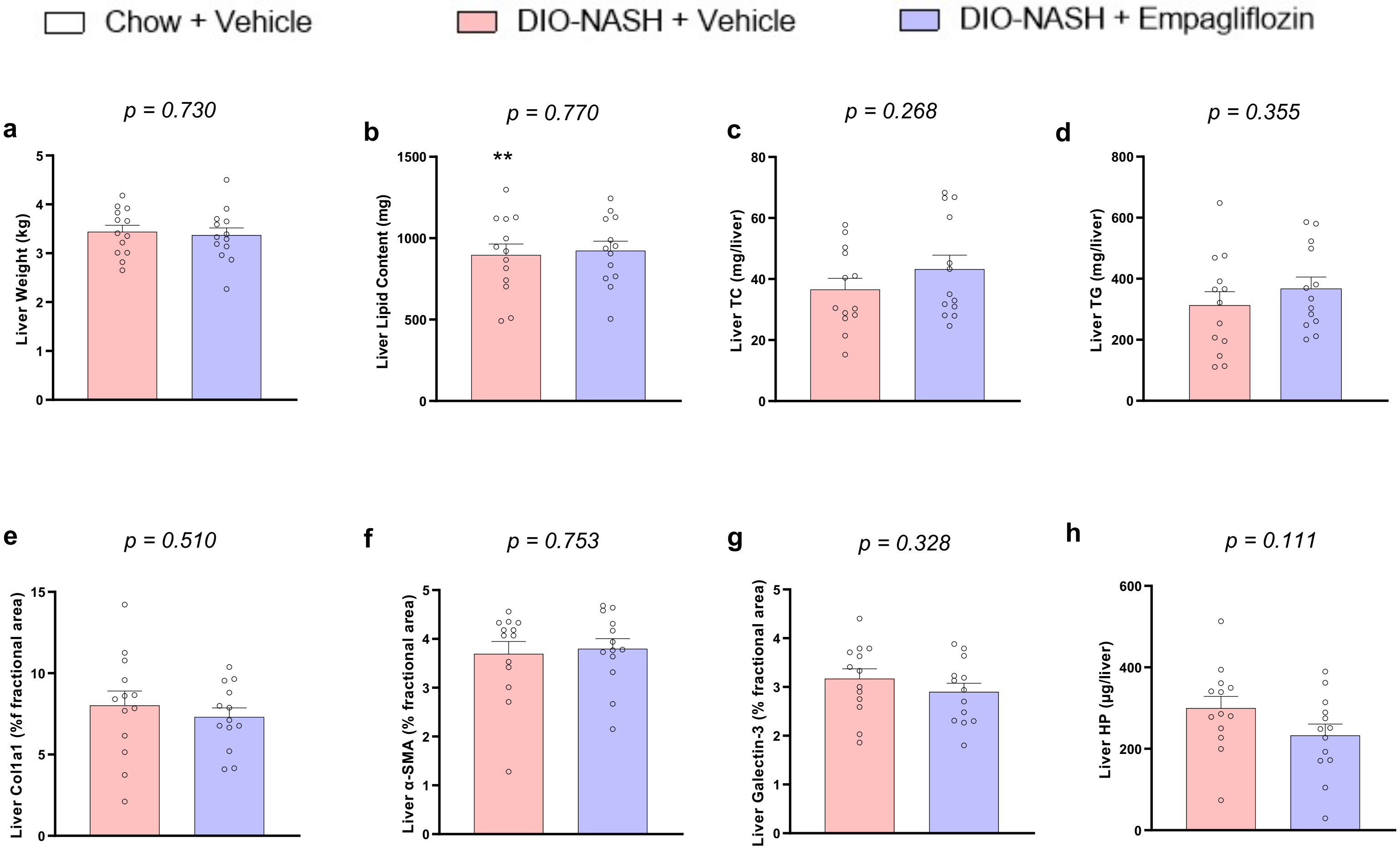

Supplemental Figure 2: Effects of empagliflozin on lipid parameters and on markers of Kupffer and stellate cell activation in the liver at treatment completion. (a) Liver weight, (b) Lipid content, (c) Total cholesterol, (d) Triglycerides; (e) Col1a1, (f) a-SMA, (g) Galectin-3 as % of liver area and (h) Hydroxyproline. Data show means ± SEMs. P from unpaired t-test is reported. Chow + vehicle n=12; DIO-NASH +Vehicle n=13; DIO-NASH + Empagliflozin n=13.

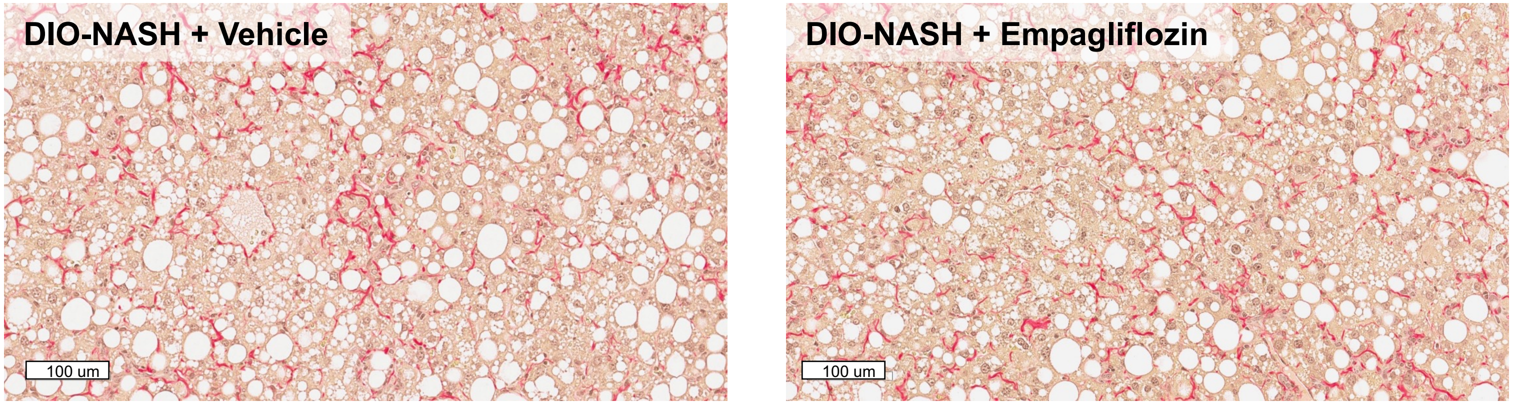

Supplemental Figure 3: Representative images of Picro Sirius red staining for liver fibrosis in DIO-NASH + Vehicle versus DIO-NASH + Empagliflozin mice.
